# Interdomain communication in a homodimeric ABC transporter

**DOI:** 10.1016/j.jbc.2024.107440

**Published:** 2024-06-05

**Authors:** Katharina-Astrid Lindt, Stefan Frühschulz, Robert Tampé, Rupert Abele

**Affiliations:** Institute of Biochemistry, Biocenter, Goethe University Frankfurt, Frankfurt, Germany

**Keywords:** ABC transporters, TAPL, interdomain signal transmission, interaction network, allosteric coupling

## Abstract

ABC transporters are found in all organisms and almost every cellular compartment. They mediate the transport of various solutes across membranes, energized by ATP binding and hydrolysis. Dysfunctions can result in severe diseases, such as cystic fibrosis or antibiotic resistance. In type IV ABC transporters, each of the two nucleotide-binding domains is connected to a transmembrane domain by two coupling helices, which are part of cytosolic loops. Although there are many structural snapshots of different conformations, the interdomain communication is still enigmatic. Therefore, we analyzed the function of three conserved charged residues in the intracytosolic loop 1 of the human homodimeric, lysosomal peptide transporter TAPL (transporter associated with antigen processing-like). Substitution of D278 in coupling helix 1 by alanine interrupted peptide transport by impeding ATP hydrolysis. Alanine substitution of R288 and D292, both localized next to the coupling helix 1 extending to transmembrane helix 3, reduced peptide transport but increased basal ATPase activity. Surprisingly, the ATPase activity of the R288A variant dropped in a peptide-dependent manner, whereas ATPase activity of wildtype and D292A was unaffected. Interestingly, R288A and D292A mutants did not differentiate between ATP and GTP in respect of hydrolysis. However, in contrast to wildtye TAPL, only ATP energized peptide transport. In sum, D278 seems to be involved in bidirectional interdomain communication mediated by network of polar interactions, whereas the two residues in the cytosolic extension of transmembrane helix 3 are involved in regulation of ATP hydrolysis, most likely by stabilization of the outward-facing conformation.

ABC transporters represent one of the largest protein superfamilies and are present in all domains of life. Driven by ATP binding and hydrolysis, ABC transporters mediate translocation of a broad range of solutes across lipid bilayers, from small molecules to lipids, peptides, and large proteins. Depending on their physiological function, they can act as importers, exporters, channels, and regulators (1, 2). Dysfunctions can be linked to severe human diseases, such as cystic fibrosis or multidrug resistance (3). Therefore, the elucidation of molecular mechanisms of this class of membrane proteins is of high interest for clinical research.

All ABC transporters share a common core architecture, composed of two conserved nucleotide-binding domains (NBDs), required for nucleotide binding and hydrolysis, and two structurally diverse transmembrane domains (TMDs), which form the substrate-binding site and the translocation pathway across the membrane (4, 5). In eukaryotes, all four domains are arranged on a single polypeptide chain, forming a full transporter, or one TMD is fused with a single NBD to a half transporter. Here, two half transporters assemble to form homodimers or heterodimers (6). In type IV ABC transporters, each TMD consists of six transmembrane helices (TMHs). The intracytosolic loops (ICLs) 1 and 2 include the cytosolic parts of TMHs 2 to 3 and 4 to 5, which extend far into the cytosol, and one connecting coupling helix (CH), respectively. The CHs are oriented parallel to the membrane plane and constitute the interaction site with the NBDs. Within the catalytic cycle, ATP-triggered dimerization of the NBDs is transmitted to the TMDs *via* the CHs, which are embedded in a groove on the interface to the NBDs. Engagement of NBDs triggers switching of the TMDs from an inward-facing (IF) to an outward-facing (OF) state, accompanied by a change from a high- to a low-affinity substrate-binding state in ABC exporters. Upon ATP hydrolysis, inorganic phosphate is released and the NBDs dissociate, which resets the transporter to the IF state (2, 5, 7, 8).

Although, an impressive number of structures of ABC transporters have been solved in recent years, there is still little known about the signal transmission between the NBD and TMD. From the perspective of the NBD, two conserved motifs are connected to the coupling helices. The Q-loop, which senses the presence of nucleotide, and the X-loop, which is exclusive to ABC type IV transporters and precedes directly the γ-phosphate sensing C-loop (9, 10, 11, 12, 13). Each TMD comprises two CHs, whereas CH1 interacts primarily with the NBD and the Q-loop in *cis*, whereas CH2 and the X-loop act in *trans* (9, 10, 14). A special characteristic of CH1 is that it also interacts directly with the amino group (N6) of the adenine base.

In this study, we focused on the transmission interface of the human half-transporter TAPL (transporter associated with antigen processing-like; ABCB9). This homodimeric transporter is localized in lysosomes and mediates ATP-dependent transport of polypeptides (6-mer up to 59-mer) from the cytosol into the lysosomal lumen and can potentially flip phospholipids from the cytosolic to the luminal leaflet of lysosomes (15, 16, 17, 18). In addition to the type IV ABC transporter core architecture, TAPL comprises an extra N-terminal 4 TMH domain, named TMD0, which is responsible for lysosomal targeting and the interaction with the lysosomal-associated membrane proteins, LAMP-1/LAMP-2. CoreTAPL forms a homodimeric complex, which is fully active in peptide transport (19, 20). Recent results demonstrated an unexpected communication between TMD and NBD, in which TAPL does not differentiate between the length of the peptides in the binding process but in the subsequent steps of transport (17). Shorter peptides are transported more efficiently than longer peptides with respect to ATP hydrolysis. TAPL exhibits a basal ATPase activity, which is not stimulated in the presence of peptides but inhibited by longer peptides. The subtle communication between TMD and NBD is also reflected in different coupling efficiencies from the nucleotide perspective. TAPL hydrolyses GTP as efficient as ATP, but peptide transport is significantly higher for ATP in comparison to GTP (17).

To study the communication between TMD and NBD, we focused on three conserved and charged residues in the ICL1 connecting TMH2 and TMH3. Mutating D278 in CH1 resulted in an inactive mutant with disrupted ATPase activity. Substitution of R288 or D292 by alanine showed subtle effects on ATP hydrolysis and peptide transport but had a drastic influence on GTP-dependent peptide transport and peptide-dependent reduction of nucleotide hydrolysis. Taking together our results, D278 seems to have a direct impact on ATP hydrolysis, whereas R288 and D292 can stabilize conformations by interacting with residues in neighboring TMHs.

## Results

### Conserved residues at the transmission interface of type IV ABC transporters

**Figure 1:**
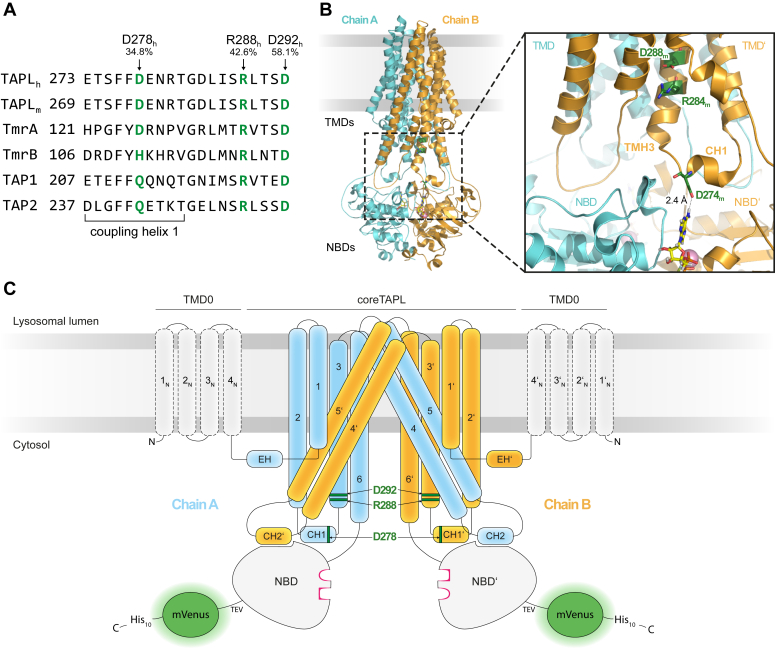
**Transmission interface of the human homodimeric ABC transporter TAPL.***A,* sequence alignment of the transmission interface of different type IV ABC polypeptide transporters (UniProt accession numbers: human TAPL [TAPL_h_], Q9NP78; mouse TAPL [TAPL_m_], Q9JJ59; *Thermus thermophilus* TmrA/B, Q72J05/Q72J04; human TAP1, Q03518; human TAP2, Q03519) performed with Clustal Omega. Residues analyzed in this study are highlighted in *green*. Identity of the depicted residues of 580 type IV ABC is given in percent. *B,* cryo-EM structure of mouse coreTAPL (Protein Data Bank code: 7V5C) (16) in complex with Mg^2+^–ADP–BeF_3_. Residues relevant for this study are labeled and depicted in *dark green*, ADP–BeF_3_ in *yellow*, and Mg^2+^ in *light pink*. Structural representation was created by PyMOL. *C,* topology model of human TAPL. The TMDs are illustrated in *blue* and *orange*, respectively. NBDs are represented as surface model, including nucleotide-binding sites (*magenta*). The C terminus of coreTAPL is fused *via* a tobacco etch virus cleavage site to an mVenus fluorescence protein and a C-terminal His_10_ tag. The four N-terminal TMHs of TMD0 (*dotted lines*) were not included in the expression construct. Residues relevant for this study are labeled and depicted in *dark green*. Domain representation does not correspond to any proportion.

To identify residues that are involved in NBD–TMD crosstalk, we performed sequence alignments of the transmission interface of 580 type IV ABC transporters from all kingdoms of life. Here, we particularly focused on ICL1, as it forms interaction networks in *cis* and *trans.* Besides the most highly conserved G283 (71.4% identity), which likely serves as a helix breaker at the N-terminal end of TMH3, we spotted three highly conserved and charged residues at the transmission interface of human TAPL: D278 (34.8% identity), R288 (42.6% identity), and D292 (58.1% identity) (Fig. 1*A*). We hypothesized that these residues potentially play a role in the communication between NBD and TMD, not only because they are highly conserved but also because of the polar interactions they form within the homodimeric transporter. D278 of human TAPL is located at the C-terminal end of CH1 at the interface between TMD and NBD (Fig. 1, *B* and *C*). Based on the cryo-EM single-particle reconstruction of coreTAPL from *Mus musculus* in its OF conformation, D278 extends toward the NBD dimer interface to interact in *trans* with Q642, which is part of the extended X-loop, and in *cis* with Y556 and the amino group at C6 of ADP trapped by beryllium fluoride (16). These interactions do not only suggest an involvement in NBD–TMD communication but also in nucleotide recognition. The residues R288 and D292 are located in the cytosolic extension of TMH3 (Fig. 1, *B* and *C*). Based on the cryo-EM reconstruction of mouse coreTAPL, R288 forms a salt bridge with E481 of TMH6 in the IF conformation, whereas D292 interacts with Q358 of TMH4 in the OF conformation. Therefore, these two residues seem to stabilize different conformations during the transport cycle. To reveal the role of D278, R288, and D292 of human TAPL in NBD–TMD crosstalk, we substituted those conserved residues with alanine in human coreTAPL, fused *via* a tobacco etch virus cleavage site to a C-terminal mVenus and His_10_ tag, to analyze the mutants by functional assays. It was established previously that the C-terminal mVenus fusion protein does not significantly alter peptide transport of coreTAPL (21). In addition, we observed comparable ATP hydrolysis rates in the presence and absence of the mVenus fusion protein (Fig. S1).

### Transmission mutants are expressed and folded correctly

**Figure 2:**
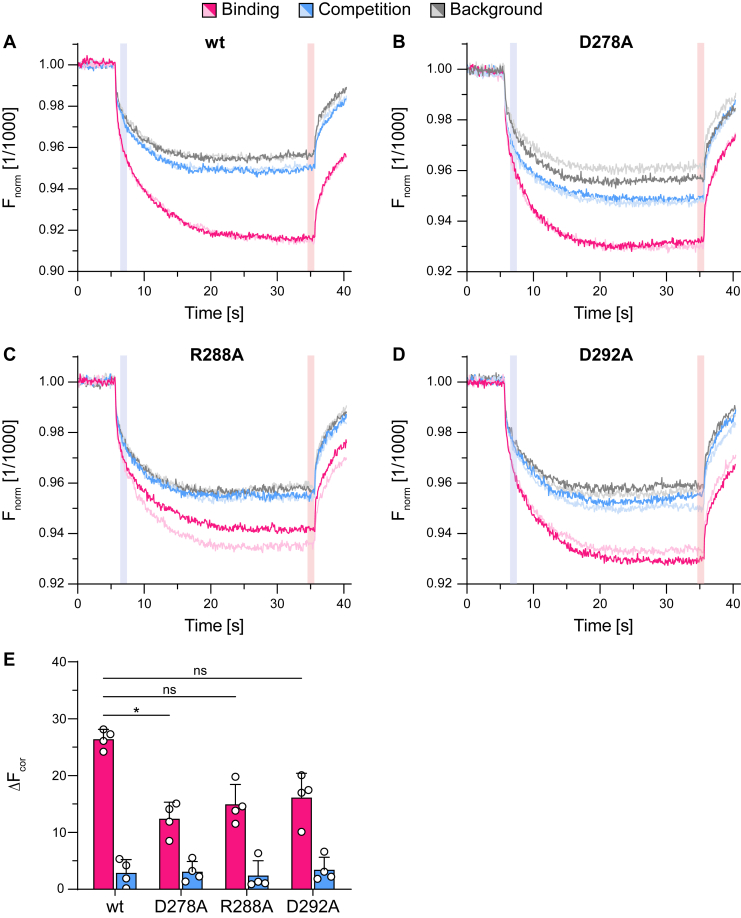
**Peptide binding of all TAPL variants indicates correct folding.***A*–*D,* thermophoretic profiles of 5 nM peptide C4^Atto-655^ that was either incubated alone (background, *gray*) or with 5 μM of GDN-purified TAPL variants in the absence (binding, *magenta*) or the presence of 150 μM competitor peptide R9L (competition, *blue*) at 22 °C (biological replicates, n = 2; technical replicates, N = 4). F_cold_ (*blue region*) at 6.2 to 7.2 s represents the intensity at the beginning of the thermophoresis, and F_hot_ (*red region*) depicts the heated state after 34.7 to 35.7 s applying 50% of infrared laser power. *E,* peptide binding and competition were determined from the difference of the thermophoretic behavior of free and bound C4^Atto-655^ between F_cold_ and F_hot_. Data represent the means and standard deviation. Statistical significances were determined using Welch’s ANOVA with post hoc Tamhane's T2 test (ns, nonsignificant, *p* > 0.05; ∗*p* ≤ 0.05; ∗∗*p* ≤ 0.01; ∗∗∗*p* ≤ 0.001; ∗∗∗∗*p* ≤ 0.0001).

To decipher the function of these conserved residues in the signal transmission between TMD and NBD, we expressed human coreTAPL (UniProt: Q9NP78, amino acids 143–766) and the aforementioned variants in *Sf21* insect cells using the baculovirus expression system (Fig. 1*C*). TAPL variants were purified by immobilized metal affinity chromatography and reconstituted into unilamellar liposomes (40:1 [w/w] lipid to protein) composed of *Escherichia coli* polar lipids and 1,2-dioleoyl-*sn*-glycero-3-phosphocholine (7:3 [w/w]). We confirmed by different methods that substitutions of D278A, R288A, or D292A do not interfere with folding and homodimeric assembly of coreTAPL: First, C-terminal mVenus as folding indicator showed a strong in-gel fluorescence indicating correct assembly of the mutants, similar to wildtype (wt) coreTAPL (Fig. S2) (22). Second, analytical size-exclusion chromatography of glyco-–diosgenin (GDN)–solubilized TAPL variants displayed a distinct peak of each variant at the same retention volume (Fig. S3). Third, the thermal stability of GDN-solubilized TAPL variants was very similar with melting temperatures between 40.9 °C and 43.7 °C (Fig. S4 and Table 1). Since TAPL with and without C-terminal mVenus fusion depicted similar melting temperature, this fluorescent fusion tag did not influence thermostability of TAPL. Finally, all TAPL variants were active in peptide binding as demonstrated by microscale thermophoresis (Fig. 2, *A*–*E*). The differences in the thermophoretic signal seem to be caused by varying thermophoretic behavior, since K_m,peptide_ are very similar for peptide transport. Taken together, we confirmed correct folding and homodimeric assembly of all TAPL variants as a prerequisite for the following experiments.

**Table 1:** Melting temperatures

Variant	T_m_ [°C]	R^2^	Statistics (n; N)
wt^w/o^	41.9 ± 0.7	0.931	2; 2
wt^mV^	43.7 ± 0.1	0.953	2; 2
D278A^mV^	41.9 ± 2.5	0.933	2; 2
R288A^mV^	41.0 ± 1.1	0.936	2; 2
D292A^mV^	40.9 ± 0.1	0.977	2; 2

n, biological replicates; N, technical replicates.

### Mutations in the transmission interface interfere differently with peptide transport

**Figure 3:**
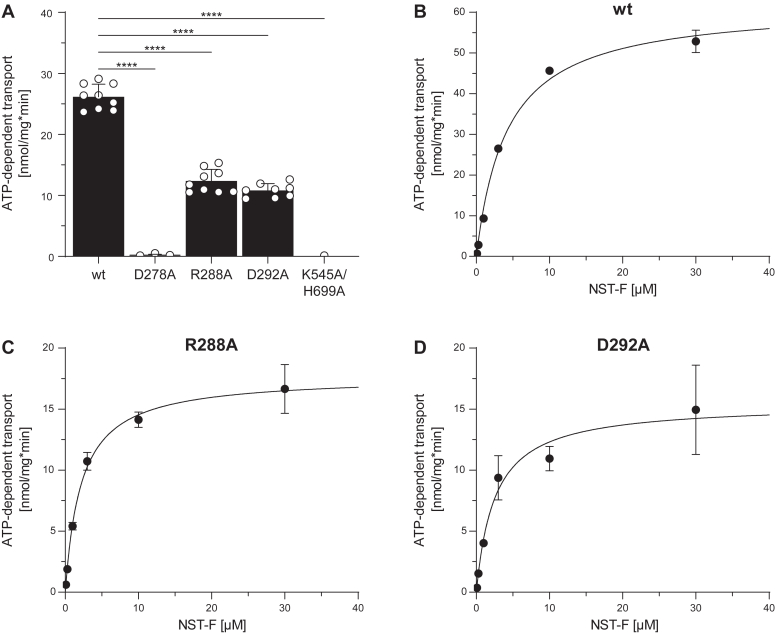
**Transmission mutants affect peptide transport differently.***A,* peptide transport was performed with 0.5 mg/ml proteoliposomes containing TAPL variants (40:1 [w/w] lipid to protein) for 15 min at 37 °C under steady-state conditions (3 mM ATP, 3 μM NST^FL^, and 5 mM MgCl_2_) including an ATP-regenerating system (biological replicates, n = 2–3; technical replicates, N = 6–9). *B–D,* peptide-dependent Michaelis–Menten kinetics were performed with 0 to 30 μM NST^FL^ for 10 min at 37 °C. Transport was quantified by fluorescence of NST^FL^ (λ_ex_/λ_em_: 480/520 nm). Presented data are normalized for equal reconstitution. Data represent the means and standard deviation. Data were fitted with the Michaelis–Menten equation. Kinetic parameters and statistics of *B*–*D* are listed in Table 2. Statistical significances were determined using Welch’s ANOVA with post hoc Tamhane's T2 test (ns, nonsignificant, *p* > 0.05; ∗*p* ≤ 0.05; ∗∗*p* ≤ 0.01; ∗∗∗*p* ≤ 0.001; and ∗∗∗∗*p* ≤ 0.0001).

To elucidate the function of these conserved residues in the transmission interface between NBD and TMD, we examined peptide transport of all TAPL variants at 3 μM of peptide NST^FL^ (RRYQNSTC^FL^L) under steady-state conditions. Strikingly, we did not observe peptide translocation of the D278A mutant, whereas R288A and D292A showed 2.5-fold decreased transport rates compared with wt TAPL. As expected, no peptide transport was detected for the K545A/H699A mutant, in which the conserved lysine and histidine of the Walker A motif and the H-loop, respectively, were substituted (Fig. 3*A*) (17). To further investigate the reason for the reduced transport of the mutants, we performed peptide-dependent Michaelis–Menten transport kinetics. D278A was excluded as no transport activity above background was detected. The Michaelis–Menten constant K_m,peptide_ of wt, R288A, and D292A were very similar, indicating that the corresponding residues are not involved in peptide binding. However, V_max_ of the mutants decreased fourfold in comparison to wt (Fig. 3, *B*–*D* and Table 2). We therefore hypothesized that the mutations R288A and D292A in TMH3 affect NBD–TMD crosstalk, whereas the transport-inactive mutant D278A in CH1 either completely disrupts the connection between NBD and TMD or interferes with ATP binding/hydrolysis due to its proximity to the nucleotide-binding site.

**Table 2:** Peptide-dependent transport kinetics

Variant	K_m,peptide_ (μM)	V_max_ (nmol/mg∗min)	R^2^	Statistics (n; N)
wt	4.1 ± 0.5	65.7 ± 2.5	0.996	2; 6
D278A	ND	ND	ND	–
R288A	2.2 ± 0.3	17.7 ± 0.5	0.983	2; 6
D292A	2.5 ± 0.7	15.4 ± 1.1	0.913	2; 6

n, biological replicates; N, technical replicates; ND, not detectable.

### Transmission mutations affect nucleotide hydrolysis

**Figure 4:**
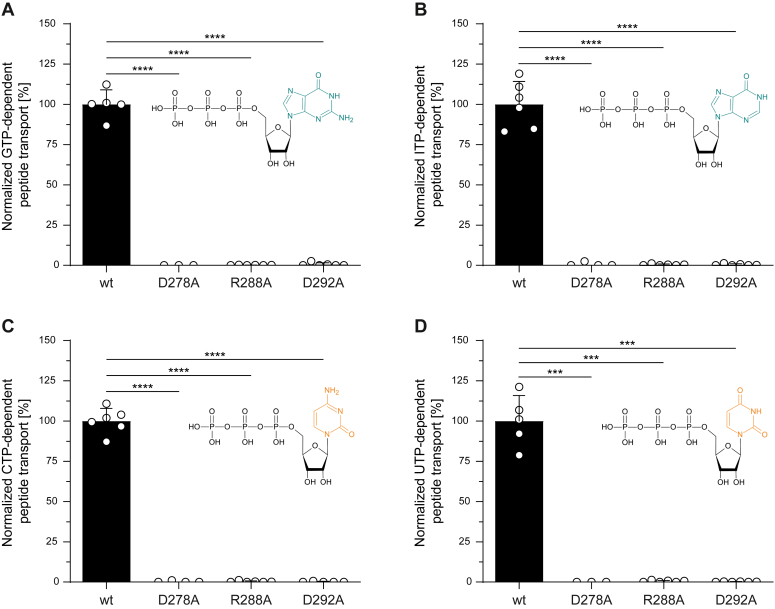
**Peptide transport of TAPL variants is NTP dependent.** Peptide transport was performed with 0.5 mg/ml proteoliposomes containing TAPL variants (40:1 [w/w] lipid to protein) for 30 min at 37 °C in the presence of 3 μM NST^FL^, 5 mM MgCl_2,_ and (*A*) 3 mM GTP, (*B*) 3 mM ITP, (*C*) 3 mM CTP, or (*D*) 3 mM UTP (biological replicates, n = 2; technical replicates, N = 5–6). Transport was quantified by fluorescence of NST^FL^ (λ_ex_/λ_em_: 480/520 nm). Presented data are normalized for equal reconstitution. Data represent the means and standard deviation. Statistical significances were determined using Welch’s ANOVA with post hoc Tamhane's T2 test (ns, nonsignificant, *p* > 0.05; ∗*p* ≤ 0.05; ∗∗*p* ≤ 0.01; ∗∗∗*p* ≤ 0.001; ∗∗∗∗*p* ≤ 0.0001).

Previous studies demonstrated that TAPL-dependent peptide transport is energized to a different extent also by other nucleotides (15). To investigate whether the mutations in the transmission interface have an impact on substrate translocation, we performed peptide transport in the presence of GTP, ITP, CTP, and UTP. Except for ATP, none of the mutants showed transport for the nucleotides studied (Fig. 4), indicating that the nucleotide itself influences NBD–TMD communication. Therefore, we further investigated whether the transmission mutants impact NTP hydrolysis.

**Figure 5:**
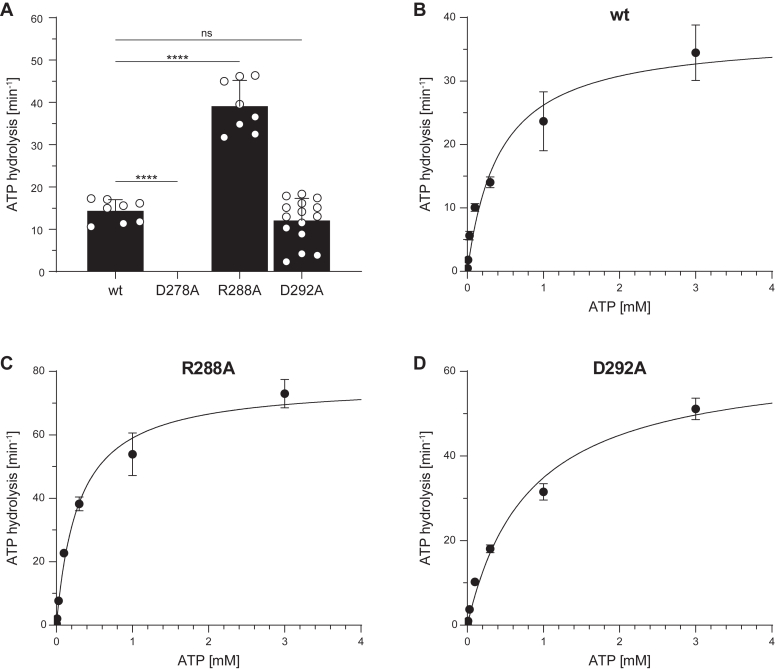
**Transport-active transmission mutants feature increased ATP turnover rates.***A,* ATP hydrolysis was analyzed with 1 mg/ml proteoliposomes containing TAPL variants (40:1 [w/w] lipid to protein) for 15 min at 37 °C under steady-state conditions (0.3 mM ATP and 5 mM MgCl_2_) (biological replicates, n = 1–5; technical replicates, N = 3–15, measured in duplicates). *B*–*D,* ATP-dependent Michaelis–Menten kinetics were performed with 0.003 to 3 mM ATP for 15 min at 37 °C. ATP hydrolysis was quantified by autoradiography. Presented data are normalized for equal reconstitution. Data represent the means and standard deviation. Data were fitted with the Michaelis–Menten equation. Kinetic parameters and statistics of *B*–*D* are listed in Table 3. Statistical significances were determined using Welch’s ANOVA with post hoc Tamhane's T2 test (ns, nonsignificant, *p* > 0.05; ∗*p* ≤ 0.05; ∗∗*p* ≤ 0.01; ∗∗∗*p* ≤ 0.001; ∗∗∗∗*p* ≤ 0.0001).

We used a sensitive radioactive-based system in which [γ-^32^P]-NTP was applied. For 300 μM of ATP, close to the K_m,ATP_ value of wt TAPL, D278A did not display any ATP hydrolysis activity, explaining the lack of transport activity of this mutant (Fig. 5*A*). For R288A, ATP hydrolysis activity was twofold increased, whereas D292A showed comparable turnover rates to wt TAPL (Fig. 5*A*). To gain further insights into ATP hydrolysis, we analyzed Michaelis–Menten kinetics for the transport-active TAPL variants. The Michaelis–Menten constants K_m,ATP_ for wt and R288A were in the range of error identical, whereas the K_m,ATP_ for D292A was twofold increased (Fig. 5, *B*–*D* and Table 3). Interestingly, the ATP turnover number k_cat_ of the R288A and D292A variants was increased 1.5- to 2-fold in comparison to wt TAPL. Taking together, the alanine substitutions of R288 and D292 have a dual effect by increasing the basal ATP hydrolysis and simultaneously reducing the transport activity.

**Table 3:** NTP-dependent hydrolysis kinetics

NTP	Variant	K_m,NTP_ (mM)	k_cat_ (min^−1^)	R^2^	Statistics (n; N)
ATP	wt	0.42 ± 0.09	37.3 ± 2.6	0.937	2; 6
	D278A	ND	ND	ND	–
	R288A	0.29 ± 0.04	76.4 ± 2.7	0.980	2; 6
	D292A	0.81 ± 0.10	63.2 ± 3.0	0.984	5; 15
GTP	wt	0.64 ± 0.06	49.4 ± 1.7	0.990	2; 6
	D278A	ND	ND	ND	–
	R288A	0.64 ± 0.06	117.4 ± 4.1	0.989	2; 6
	D292A	1.99 ± 0.47	61.6 ± 7.2	0.969	2; 6

n, biological replicates; N, technical replicates, measured in duplicates; ND, not detectable.

**Figure 6:**
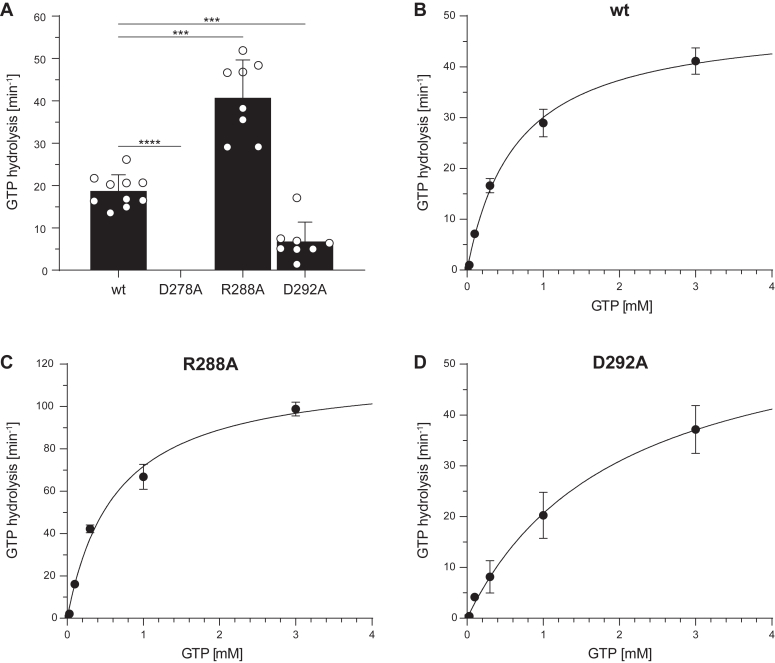
**Transport-active TAPL variants hydrolyze GTP similar to ATP.***A,* GTP hydrolysis was analyzed with 1 mg/ml proteoliposomes containing TAPL variants (40:1 [w/w] lipid to protein) for 15 min at 37 °C under steady-state conditions (0.3 mM GTP, 5 mM MgCl_2_) (biological replicates, n = 1–4; technical replicates, N = 3–10, measured in duplicates). *B*–*D,* GTP-dependent Michaelis–Menten kinetics were performed with 0.003 to 3 mM GTP for 15 min at 37 °C. GTP hydrolysis was quantified by autoradiography. Presented data are normalized for equal reconstitution. Data represent the means and standard deviation. Data were fitted with the Michaelis–Menten equation. Kinetic parameters and statistics of *B*–*D* are listed in Table 3. Statistical significances were determined using Welch’s ANOVA with post hoc Tamhane's T2 test (ns, nonsignificant, *p* > 0.05; ∗*p* ≤ 0.05; ∗∗*p* ≤ 0.01; ∗∗∗*p* ≤ 0.001; ∗∗∗∗*p* ≤ 0.0001).

Next, we checked GTP hydrolysis of all TAPL variants. Although the mutants are inactive in GTP-dependent peptide transport, wt, R288A, and D292A hydrolyzed GTP (300 μM) similar as ATP, whereas D278A was inactive (Fig. 6*A*). The Michaelis–Menten constant K_m,GTP_ for wt and R288A was identical, whereas D292A displayed an increased K_m,GTP_ value (Fig. 6, *B*–*D* and Table 3). As for ATP, the GTP turnover number k_cat_ for R288A increased twofold in comparison to wt TAPL, whereas k_cat_ of D292A was slightly elevated. In summary, the D278A substitution rendered coreTAPL inactive in nucleotide hydrolysis; the mutations R288A and D292A, however, accelerated the basal nucleotide hydrolysis activity, whereas only ATP energized peptide transport.

### Mutations in the transmission interface do not affect ATP binding

**Figure 7:**
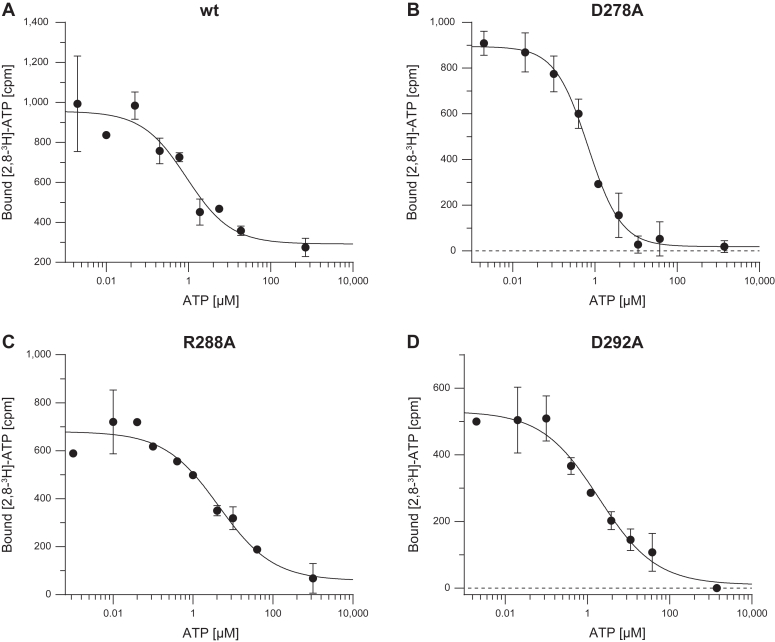
**TAPL transmission mutants do not affect ATP binding.** About 0.5 μM GDN-purified TAPL was incubated with 0.2 μM [2,8-^3^H]-ATP in the presence of 5 mg/ml nanoSPA beads and increasing concentrations of nonlabeled ATP (0–1000 μM) for 2 h at 4 °C. *A,* ATP binding of wt, (*B*) D278A, (*C*) R288A, and (*D*) D292A. ATP binding was quantified by monitoring *blue* light emission of the nanoSPA beads. Background-corrected data were fitted with an equation for one-site competition with variable Hill slope. IC_50_ values and statistics of two experiments are listed in Table 4. Data represent the means and standard deviation.

As neither peptide transport nor NTP hydrolysis for D278A was observed, we analyzed ATP binding of all TAPL constructs by scintillation proximity assays (SPAs), which allow interaction analysis of ligands with low affinity and fast dissociation rates. GDN-solubilized TAPL was incubated with nanoSPA beads in the presence of tracer amounts of [2,8]-^3^H-ATP and increasing concentrations of nonlabeled ATP (0–40 μM) as a competitor. The amount of TAPL-bound radioactive ATP was determined by scintillation counting (Fig. 7). The IC_50_ values of all transmission mutants are comparable to wt (Table 4), confirming that none of the mutations affect ATP binding. In conclusion, the lack of ATP hydrolysis of the D278A mutant is rather due to an impaired signal transmission between NBDs to TMDs than an impaired nucleotide binding.

**Table 4:** ATP binding

Variant	IC_50_ (μM)	R^2^	Statistics (n; N)
wt	1.9 ± 1.3	0.943	2; 4
D278A	0.9 ± 0.2	0.980	2; 4
R288A	5.6 ± 1.7	0.935	2; 4
D292A	1.3 ± 0.8	0.949	2; 4

n, biological replicates; N, technical replicates.

### Downregulation of ATP hydrolysis by peptide transport

**Figure 8:**
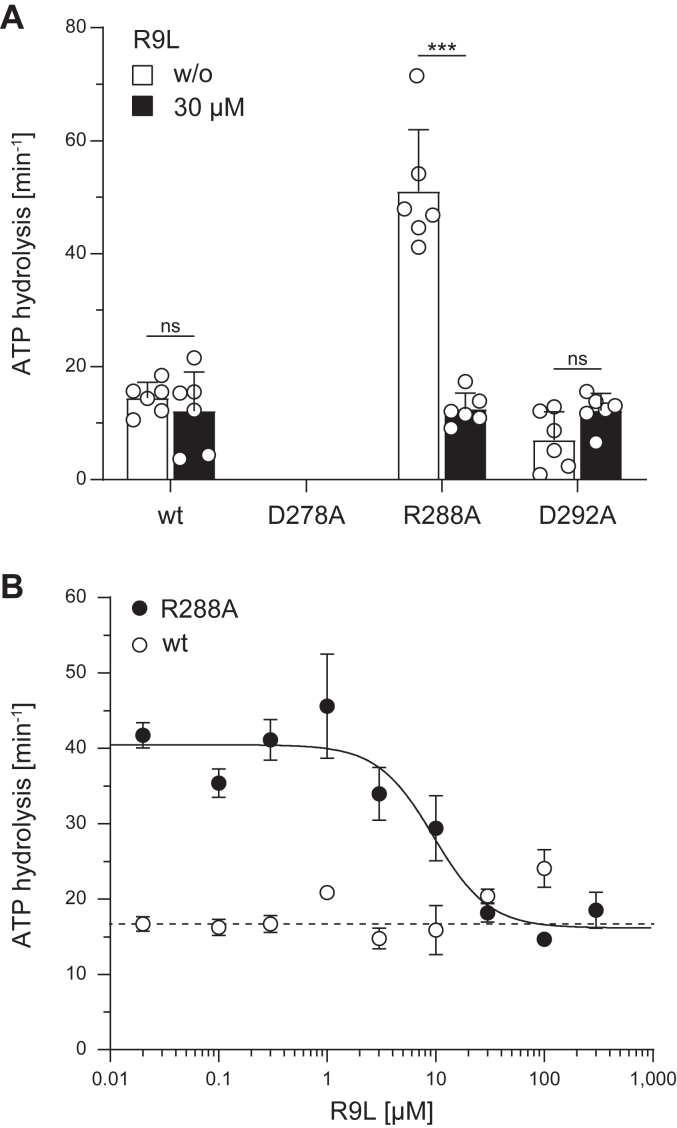
**ATP hydrolysis of R288A is substrate dependent.** ATP hydrolysis was analyzed with 1 mg/ml proteoliposomes containing TAPL variants (40:1 [w/w] lipid to protein) for 15 min at 37 °C under steady-state conditions (0.3 mM ATP, 5 mM MgCl_2_). *A,* ATP hydrolysis rates of TAPL variants in the absence (*white*) and presence of 30 μM R9L (*black*) (biological replicates, n = 2; technical replicates, N = 6). *B,* ATP hydrolysis rates of wt (*white*) and R288A (*black*) dependent on peptide R9L concentrations (0–300 μM) (biological replicates, n = 1; technical replicates, N = 3). ATP hydrolysis was quantified by autoradiography. Presented data are background corrected (*A*, in the presence of 0.5 mM vanadate as TAPL-specific inhibitor; *B*, autohydrolysis) and normalized for equal reconstitution. Data were fitted with an equation for one-site competition with variable Hill slope, resulting in an IC_50_ value of 9.5 μM for R288A. Data represent the means and standard deviation. Statistical significances were determined using a parametric, unpaired, two-tailed *t* test with Welch’s correction (ns, nonsignificant, *p* > 0.05; ∗*p* ≤ 0.05; ∗∗*p* ≤ 0.01; ∗∗∗*p* ≤ 0.001; ∗∗∗∗*p* ≤ 0.0001).

To investigate the impaired communication between NBD and TMD of the mutants, we compared basal and peptide-induced ATP hydrolysis rates under saturating peptide concentrations (30 μM). Previous studies demonstrated for wt TAPL that ATP hydrolysis decreased with increasing concentrations of longer peptides (16–18-mer peptides) but was unaffected in the presence and absence of a 9-mer peptide (17). As expected, we reported very similar ATP hydrolysis rates for wt TAPL in the presence and absence of 30 μM R9L. As previously observed, ATP hydrolysis of D278A was not detectable. In addition, ATP hydrolysis of D292A was not affected by adding the 9-mer peptide. However, to our surprise, ATP hydrolysis of R288A was reduced in the presence of the 9-mer peptide to the level of wt TAPL (Fig. 8*A*). This was also reflected in Michaelis–Menten kinetics in the presence of saturating R9L concentration (150 μM) where the turnover number (k_cat_) of wt and D292A was identical to the basal ATPase activity, whereas the k_cat_ of R288A showed a reduction of the R9L to wt level. Importantly, the Michaelis–Menten constant K_m,ATP_ did not change in the presence of saturating peptide concentrations for all TAPL variants (Fig. S5). To analyze the reduction of ATP hydrolysis of R288A in more detail, peptide-dependent ATP hydrolysis was investigated. For wt, the ATP hydrolysis was independent of the added peptide, whereas the ATP hydrolysis rate of R288A decreased in a peptide-dependent manner, with an IC_50_ value of 9.5 μM (Fig. 8*B*), correlating with the peptide affinity. Interestingly, at saturating peptide concentrations, the hydrolysis rates of wt and R288A are almost identical (Fig. 8*B*). In conclusion, ATP hydrolysis of wt and D292A is not influenced by the presence of the 9-mer peptide, whereas ATPase activity of R288A is decreased in the presence of peptide, implying a sensing of the peptide.

## Discussion

In this work, we identified three conserved and charged residues (D278, R288, and D292) at the transmission interface of the homodimeric type IV ABC transporter TAPL, which differ in NBD–TMD communication. The D278A substitution impaired ATP hydrolysis and peptide translocation but still allowed peptide and ATP binding as wt TAPL. Interestingly, substitution of Q277 in TAP1 (homolog to D278 in hTAPL) with cysteine did not influence peptide binding and transport of the heterodimeric antigen transport complex TAP, although this residue is in proximity to the X-loop of TAP2 (*trans* NBD in TAPL) as demonstrated by cysteine crosslinking (11). However, the X- and C-loop of TAP2 are part of the degenerate ATP-binding site of the heterodimeric TAP complex, and, therefore, Q277 does not seem to be essential for interdomain communication, or a cysteine substitution in contrast to an alanine substitution is tolerated. In contrast, the homodimeric TAPL transporter comprises two active nucleotide-binding sites, and therefore, mutation of D278A impairs peptide transport. Recent structural data of mouse TAPL in the ADP–BeF_3_-bound OF state showed that this aspartate residue interacts with the amino group of the C6 atom of ADP–BeF_3._ It extends toward the NBD dimer interface where it interacts in *cis* with the highly conserved Y552 (52.8%, Y556 of hTAPL) in the NBD of the same subunit and in *trans* with Q638 (Q642 in hTAPL) in the NBD of the other subunit, directly upstream of the signature motif and part of the extended X-loop (Protein Data Bank [PDB] code: 7V5C) (16). Moreover, one residue upstream of D274, E275 (E279 in hTAPL) forms a polar interaction with R510 (R514 in hTAPL), which is in direct neighborhood of Y509 (Y513 in hTAPL), being part of the A-loop and forming a π–π interaction with adenine. Similar interaction networks were observed in other type IV ABC transporters such as TmrAB and CFTR (23, 24). We hypothesize that D278 is part of an interaction network of residues from the TMD and the NBD including the bound nucleotide. Removing the carboxyl group of D278 leads to a collapse of this interdomain interaction network causing small structural rearrangements, which interfere with ATP hydrolysis. D278 is therefore a prime example of how a mutation in the TMD of an ABC transporter can influence ATP hydrolysis in the NBD and reflect the allosteric coupling between both domains. Although aspartate is the most frequent amino acid at the C-terminal end of CH1 of type IV ABC transporter, a hydrophobic residue is occasionally found at this position (*e.g.*, Sav1866, PDB code: 2HYD, (10)). Thus, differences in allosteric coupling might exist, which are also reflected in the coupling efficiency between ATP hydrolysis and peptide translocation. The heterodimeric TAP transporter features an allosteric coupling between peptide transport and ATP hydrolysis with no basal ATPase activity (25), whereas the homodimeric TAPL transporter shows a basal ATPase activity, which is not increased by peptide translocation (17).

The transmission mutants R288A and D292A showed both a reduced ATP-dependent peptide transport activity compared to wt TAPL, whereas basal ATPase activity was even higher as wt TAPL. Derived from the cryo-EM structure of mouse TAPL, both residues are located on the cytosolic extension of TMH3 and are separated by one helical turn. In the IF conformation, both residues form polar contacts with residues of TMH6 of the same subunit. After conversion to the OF conformation, the electrostatic interactions between R284 (R288 in hTAPL) in TMH3 and E477 (E481 in hTAPL) in TMH6 are broken, whereas a H-bond between D288 (D292 in hTAPL) and Q354 (Q358 hTAPL) is formed. Comparable interactions were also observed in TmrAB (PDB code: 6RAF, 6RAH) (23) and Sav1866 (PDB code: 2HYD) (10). Substitution of either of these residues by alanine weakens the interaction of TMH3 with TMH6 in the IF conformation and with TMH4 in the OF conformation, leading to an increased Gibbs-free energy of the IF or OF conformation, respectively, without changing the energy level of the transition states. Therefore, we suggest that this reduced conformational stability lowers the activation energy so that the transporter can switch faster between both conformations, which is reflected in a higher basal ATPase activity of the TAPL transmission mutants R288A and D292A in comparison to wt TAPL. Equivalent mutations of R136 (R288 in hTAPL) and D140 (D292 in hTAPL) in TmrA of the heterodimeric ABC transporter TmrAB resulted also in an increased ATP hydrolysis rate. In addition, cross-linking experiments of the same mutants revealed that in the ATP–vanadate-trapped state, most of the transporter complexes are not found in the OF_open_ conformation, as observed for wt TmrAB, but in the OF_occluded_ state, which supports our hypothesis that the OF state is short lived (26).

Interestingly, the higher basal ATPase activity of R288A and D292A does not come along with higher peptide transport rates but with lower transport compared to wt TAPL. A lower transport rate was also reported for the single cysteine mutant R287C of TAP1 (R288 in hTAPL) (25). Two scenarios are conceivable for how the faster switching of the conformations affects the transport rate. First, the peptide transport of the mutants can be reduced since the decay of the IF conformation is faster than the association rate of the peptide to TAPL. This would induce futile ATPase cycles. In this case, the K_m,peptide_ should be increased in comparison to wt TAPL, which we did not observe. In the second scenario, the dwell time in the OF conformation of the mutants is shortened, and therefore, release of peptide on the luminal site is reduced. For the R288A mutant, an additional level of complexity exists since ATP hydrolysis decreased in a 9-mer peptide concentration-dependent manner. A reduction of ATP hydrolysis was also found for wt TAPL but only if longer peptides (16–18-mer) were applied (17). In this case, it would be possible that the peptide sequence between both ends bulges into the lumen of the binding pocket, and the rearrangement during the conformational change slows down the transport and thus also ATP hydrolysis. For R288A, we speculate that the peptide drives the transporter through an entire transport cycle, which needs more time than the futile cycles of the mutant. This idea is supported by the observation that TAPL is inhibited in *trans* by high luminal peptide concentrations and depicts unidirectional transport (21), meaning as far as peptide-bound TAPL enters the occluded state, the peptide has to be released on the luminal site to return *via* a peptide-free occluded state to the IF conformation.

Notably, peptide transport of wt TAPL was energized by all nucleotide triphosphates tested, Hydrolysis of ATP and GTP was identical highlighting the importance of the hydrogen bond between the amino group at C6 of adenine and D278 for the communication between the nucleotide-binding site and the TMD. In conclusion, this hydrogen bond seems to transmit conformational changes in the NBD to the TMD. The additional interactions of D278 with residues in the NBD ensure correct arrangement of the catalytic center for ATP hydrolysis. Although NTP hydrolysis was not affected, the impaired peptide transport of R288A and D292A for other NTPs than ATP can result from a synergistic effect between destabilization of the OF conformation in conjunction with the impaired transmission of conformation changes between NBD and TMD.

In summary, we have analyzed the function of three conserved and charged residues in the ICL1 of a homodimeric type IV ABC transporter. We demonstrated that the aspartate residue at the C-terminal end of CH1 is essential for ATP hydrolysis and peptide transport. Most likely, the function of D278 is to stabilize an interaction network comprising residues from both NBDs and the bound nucleotide and transmitting changes bidirectionally between TMD and NBD. The two charged residues in the cytosolic extension of TMH3 are involved in the regulation of ATP hydrolysis presumably by stabilizing the OF conformation through polar interactions with TMH4. In conclusion, these three residues are part of an allosteric network, which regulates coupling and uncoupling of ATP hydrolysis with peptide transport.

## Experimental procedures

### Constructs and cloning

Human coreTAPL (UniProt: Q9NP78, amino acids 143–766) in the presence or the absence of a C-terminal mVenus fusion protein was fused to a C-terminal His_10_ tag and cloned into pFastBac1 (21). TAPL mutants D278A, R288A, D292A, and K545/H699A of the mVenus fusion construct (numbering refers to full-length human TAPL) were generated by site-directed mutagenesis using the primer pairs D278A 5′-CTTCTTTGCTGAGAACCGCACAGG-3′ and 5′-GTTCTCAGCAAAGAAGCTTGTCTC-3′, R288A 5′-CCTCATCTCCGCCCTGACCTCGGACAC-3, and 5′-TCCCCTGTGCGGTTCTCA-3′, D292A 5′-CTCGGCCACCACCATGGTC-3′ and 5′-CATGGTGGTGGCCGAGGTCAG-3′, K545A 5′-CCTCGGGCAGTGGGGCTAGCTCCTGTG-3′ and 5′-GGATGTTGACACAGGAGCTAGCCCCACTGC-3′, H699A 5′-CACGGTACTCATCATCGCGGCTCGGCTGAGCAC-3′ and 5′-GTGCTCCACGGTGCTCAGCCGAGCCGCG ATGATG-3’ (mutated nucleotides are underlined). Successful mutagenesis was verified by DNA sequencing. Heat-shock transformation of the cloned constructs was performed in *E. coli* One Shot Mach1 T1 (Thermo Scientific) for amplification of the constructs and *E. coli* DH10α for blue-white screening. Afterward, *Sf21* insect cells were transfected with bacmid DNA to produce the V_0_ virus. About 2 × 3 ml cell suspension with a cell density of 0.25 × 10^6^ cells/ml in Sf-900 II SFM medium (Gibco) were seeded into two wells of a 6-well plate. The cells were allowed to attach to the bottom of the well for 15 min before transfection. About 40 μl bacmid DNA were mixed with 180 μl culture medium, and 20 μl X-tremeGENE HP DNA transfection reagent (Roche/Merck) was mixed with 80 μl culture medium. Both mixtures were combined and incubated at room temperature for 5 to 10 min. After incubation, 150 μl transfection mixture was added dropwise to each well, and the cells were incubated at 29 °C for 72 h. After 3 days, the supernatant containing the V_0_ virus was collected. For virus amplification, 250 ml of *Sf21* insect cells with a cell density of 1 × 10^6^ cells/ml were infected with 5 ml V_0_ virus. The cell density was maintained at 1 × 10^6^ cells/ml until the day of proliferation arrest. The baculovirus-infected insect cells (BIICs) were harvested at 800 rpm for 5 to 10 min. For cryoconservation, the BIICs were resuspended in cryomedium (Sf-900 II SFM, 10% dimethyl sulfoxide [Sigma–Aldrich/Merck], 10 mg/ml bovine serum albumin [Roth], sterile filtered) at a cell density of 1 × 10^7^ cells/ml. 1 ml aliquots was frozen at −80 °C.

### Expression, purification, and reconstitution

Large-scale expression of TAPL variants was carried out in *Sf21* insect cells. About 375 ml of *Sf21* insect cells with a density of 1 × 10^6^ cells/ml were infected with 5 × 10^6^ BIICs. The cell density was maintained at 1 × 10^6^ cells/ml until proliferation arrest. mVenus fluorescence was monitored daily, using a microplate reader (CLARIOstar; BMG LABTECH) at λ_ex/em_ = 515/530 nm, until it reached a plateau (4–5 days postinfection). The cells were harvested at 1500*g* for 10 min. The cell pellet was stored at −20 °C. For protein purification, the cells were thawed on ice and lysed with 40 strokes of a tight-fitting Dounce homogenizer (Wheaton) in the presence of 10 mM Tris–HCl (pH 7.5) and 1× protease inhibitors (HP mix; Serva Electrophoresis GmbH). Cell debris and nuclei were removed by centrifugation at 200*g* for 4 min, followed by 700*g* for 8 min, at 4 °C. Crude membranes were spun down at 40,000*g* for 30 min at 4 °C. Depending on the following experiment, crude membranes (5 mg/ml total protein) were either solubilized with 1% (w/v) of dodecyl-β-d-maltoside (DDM; Carl Roth) or GDN (Anatrace) for 1 h at 4 °C. The solubilizate was incubated with 1 ml bed volume of nickel–nitrilotriacetic acid beads (Qiagen) for 1 h at 4 °C. Subsequently, the beads were washed with 3x 10 bed volumes of washing buffer (20 mM Hepes, 140 mM NaCl, 20 mM imidazole, 15% [v/v] glycerol, 0.05% [w/v] DDM, or 0.1% [w/v] GDN, pH 7.5), followed by elution with 500 mM imidazole (washing buffer containing 500 mM imidazole). In case samples were designated for reconstitution into liposomes, the buffer was exchanged with PD-10 desalting columns (Cytiva) (20 mM Hepes, 140 mM NaCl, 15% [v/v] glycerol, 0.05% [w/v] DDM, 1x protease inhibitors [HP mix, Serva Electrophoresis GmbH], pH 7.5) after affinity chromatography. For liposome preparation, *E. coli* polar lipids and 1,2-dioleoyl-*sn*-glycero-3-phosphocholine (Avanti Polar Lipids) dissolved in chloroform were mixed in a 7:3 (w/w) ratio, and the solvent was removed by vacuum evaporation. Afterward, lipids were resuspended in reconstitution buffer (20 mM Hepes, 140 mM NaCl, 5% [v/v] glycerol, pH 7.5) to reach a final concentration of 10 mg/ml, followed by sonication for 30 min at 35 °C and five freeze–thaw cycles in liquid nitrogen. Before reconstitution, liposomes were extruded with a 400 nm polycarbonate filter, diluted to 2.5 mg/ml of lipids, and destabilized with Triton X-100 for 30 min. TAPL was added in a 1:40 (w/w) protein to lipid ratio and incubated on an overhead shaker for 30 min at 4 °C. To remove detergent, 2x 40 mg Bio-Beads SM-2 resin (Bio-Rad) were added 30 min and 1 h postreconstitution, and additional 2x 80 mg Bio-Beads were added 12 h and 14 h postreconstitution. Proteoliposomes were pelleted at 2,67,000*g* for 30 min at 4 °C and resuspended in reconstitution buffer. Aliquots were snap-frozen in liquid nitrogen and stored at −80 °C. To normalize reconstitution efficiency of all TAPL variants, proteoliposomes were loaded on a precast protein gel (Any kD Mini-PROTEAN TGX Precast Protein Gels; Bio-Rad) in nonreducing SDS-loading buffer (0.004% [w/v] bromphenol blue, 6% [v/v] glycerol, 2% [w/v] SDS, 50 mM Tris–HCl, pH 6.8). The amount of mVenus fluorescence per lane was quantified by in-gel fluorescence at λ_ex/em_ = 480/535 nm (Fusion FX; Vilber) and analyzed by ImageJ (27).

### Size-exclusion chromatography

Analytical size-exclusion chromatography runs of TAPL variants were performed with an ÄKTA pure micro system (Cytiva), equipped with a Superose 6 Increase 3.2/300 (2.4 ml bed volume [Cytiva]) equilibrated in running buffer (20 mM Hepes, 140 mM NaCl, 100 mM imidazole, 15% [v/v] glycerol, 0.1% [w/v] GDN, pH 7.5). About 25 μl of GDN-purified TAPL in GDN-containing elution buffer were injected on the column. A flow rate of 0.04 ml/min was applied at 4 °C. Detectors monitored absorbances of 280 and 515 nm.

### Peptide synthesis, labeling, and purification

Peptides (RRYQNSTCL [NST], RRYQKSTEL [R9L], and RRYCKSTEL [C4]) were synthesized by automated microwave-assisted solid-phase peptide synthesis by Fmoc chemistry (CEM; Liberty Blue). NST was labeled with five-iodoacetamide fluorescein (Sigma–Aldrich), whereas C4 was labeled with Atto-655 iodoacetamide (Atto-Tec). Peptide and dye were mixed in a 1:2 M ratio in PBS/*N*,*N*-dimethylformamide (8.1 mM Na_2_HPO_4_, 1.8 mM KH_2_PO_4_, 137 mM NaCl, 2.7 mM KCl, 33% [v/v] *N*,*N*-dimethylformamide, pH 6.5) and incubated for 2 h at 20 °C with vigorous stirring. Labeled peptides were purified by reversed-phase C18 high-pressure liquid chromatography, using a linear water/acetonitrile gradient from 5 to 60% (v/v) supplemented with 0.1% trifluoroacetic acid. Solvent was removed by lyophilization. Peptide identity was confirmed by liquid chromatography–mass spectrometry (Waters BioAccord LC–MS): RRYQNSTC^FL^L (NST^FL^, M_calc_: 1527.632 Da, M_obs_: 1527.623 Da), R9L (M_calc_: 1179.636 Da, M_obs_: 1179.642 Da), and RRYC^Atto-655^KSTEL (C4^Atto-655^, M_calc_: 1803.843 Da, M_obs_: 1803.845 Da).

### Peptide binding

Peptide binding of GDN-purified TAPL variants was analyzed by microscale thermophoresis on a Monolith NT.155Pico (NanoTemper Technologies) at 22 °C. About 5 nM of C4^Atto-655^ were incubated either alone (background), with TAPL (5 μM, binding), or TAPL (5 μM) and unlabeled R9L (150 μM, 3 × 10^4^-fold excess for competition), respectively, in GDN-containing elution buffer. Samples (6 μl) were transferred into hydrophilic capillaries (Monolith Premium Capillaries) and heated for 30 s by the infrared laser (power 50%). Thermophoretic fluorescence changes of the C4^Atto-655^ peptide were detected using 3% LED power for excitation (λ_ex/em_: 598–642 nm/657–720 nm). Fluorescence change ΔF was calculated from ratio of the mean intensities at the beginning (6.2–7.2 s, F_hot_) and at the end of heating (34.7–35.7 s, F_cold_):

(1) $$\Delta F=\frac{F_{\text{hot}}}{F_{\text{cold}}}$$

ΔF in the absence of TAPL (background) was subtracted from the ΔF in the presence of TAPL (binding/competition) and depicted as ΔF_cor_.

(2) $${\Delta F}_{cor}=\Delta F\left(\text{binding}\;\text{or}\;\text{competition}\right)-\Delta F\left(\text{background}\right)$$

### Thermal stability

The thermal stability of GDN-purified TAPL variants was determined according to the cellular thermal shift assay (28). For this, 60 μl of 0.4 μM purified TAPL variants in 20 mM Hepes, 140 mM NaCl, 0.05% (w/v) GDN, pH 7.5 were incubated at 15 to 60 °C for 5 min. To separate aggregates from soluble TAPL, samples were centrifuged for 15 min at 4,30,000*g* and 4 °C. About 40 μl of the supernatant were supplemented with nonreducing SDS-loading buffer (0.004% [w/v] bromphenol blue, 6% [v/v] glycerol, 2% [w/v] SDS, 50 mM Tris–HCl, pH 6.8). About 15 μl were loaded on a 10% polyacrylamide SDS gel and separated by applying for 15 min a voltage of 80 V followed by 120 V for 1 h. Subsequently, the proteins were transferred onto polyvinylidene fluoride membrane (Carl Roth; 0.45 μM pore size) and visualized by immunodetection using monoclonal mouse anti-His-tag antibody (Merck; H1029) followed by goat antimouse antibody coupled to horseradish peroxidase (Merck; A2554). The chemiluminescent signal was quantified (Vilber Fusion FX) and analyzed by ImageJ (27). The apparent melting temperatures (*T*_m_) were calculated by plotting the normalized intensity against temperature and fitting the data by a sigmoidal dose–response equation with variable Hill slope by GraphPad Prism software (GraphPad Software).

### Peptide transport

Peptide transport of reconstituted TAPL variants was performed with 50 μl of proteoliposomes (0.5 mg/ml lipids) in transport buffer (20 mM Hepes, 107 mM NaCl, 3 mM MgCl_2_, 5% [v/v] glycerol, pH 7.5) in the presence of 3 μM NST^FL^ and 3 mM NTP, if not stated differently. For Michaelis–Menten kinetics, an ATP-regenerating system consisting of 0.5 mg/ml creatine kinase and 10 mM creatine phosphate was applied. Transport was started by adding NTP, followed by incubation at 37 °C for 15 min. To determine background signal, transport was performed in transport buffer without NTP. The process was stopped by adding 200 μl ice-cold stop buffer (PBS, 10 mM EDTA, pH 7.5). Proteoliposomes were transferred onto microfilter plates (MultiScreen filter plates with Durapore membrane, 0.65 μm pore size; Merck) and washed three times with 250 μl ice-cold stop buffer before proteoliposomes were lysed with 250 μl lysis buffer (PBS, 1% SDS, pH 7.5). To eliminate fluorescence of the mVenus fusion protein, samples were heated to 95 °C for 10 min. About 200 μl were transferred to a black, F-BOTTOM 96-well plate (Greiner), and the amount of transported peptide was determined with a microplate reader (CLARIOstar) at λ_ex/em_ = 483/530 nm. Peptide-dependent transport kinetics were determined by analyzing background-corrected data by Michaelis–Menten kinetics:

(3) $$v=V_{\max}\;\times \;\frac{\left[S\right]}{K_{m,p\text{eptide}}\;+\;\left[S\right]}$$

Here, v represents the concentration-dependent transport rate, V_max_ the maximum transport rate, [S] the substrate concentration, and K_m,peptide_ the peptide-dependent Michaelis–Menten constant.

### NTP hydrolysis

GTP and ATP hydrolysis of reconstituted TAPL variants were performed in 10 μl NTPase buffer (20 mM Hepes, 107 mM NaCl, 5 mM NaN_3_, 1 mM ouabain, 50 μM EGTA, 5 mM MgCl_2_, 5% [v/v] glycerol, pH 7.5) containing proteoliposomes (1 mg/ml lipids) and NTP supplemented with tracer amounts of [γ-^32^P]-NTP (Hartmann Analytics) in the presence or the absence of R9L peptide (RRYQSTEL) for 15 min at 37 °C. Samples designated for autohydrolysis did not contain proteoliposomes. About 1 μl of the sample was spotted onto TLC plates (TLC PEI Cellulose-F; Merck), and inorganic phosphate was separated from nonhydrolyzed NTP by thin layer chromatography using an equimolar mixture (0.8 M) of LiCl and acetic acid. ^32^P_i_ and [γ-^32^P]-NTP were determined by autoradiography (PMI-Personal Molecular Imager; Bio-Rad) and quantified by ImageJ (27). The amount of hydrolyzed NTP was calculated by the ratio of both signals. Data were corrected for autohydrolysis, if not stated differently, and NTP-dependent Michaelis–Menten kinetics were determined according to Equation 3, with v representing the concentration-dependent hydrolysis rate, V_max_ the maximum hydrolysis rate, [S] the NTP concentration, and K_m,NTP_ the nucleotide-dependent Michaelis–Menten constant.

### ATP binding

ATP binding of GDN-solubilized TAPL was analyzed by SPAs. In a total volume of 115 μl, 0.7 μM TAPL was preincubated with 0.3 μM [2,8-^3^H]-ATP (PerkinElmer) and increasing concentrations of nonlabeled ATP (0–5.3 mM; Sigma–Aldrich) in binding buffer (20 mM Hepes, 140 mM NaCl, 10 mM MgCl_2_, 5% [v/v] glycerol, 0.05% [w/v] GDN, pH 7.5). After 1 h at 4 °C, 52.5 μl of the preincubated TAPL–ATP mix was transferred onto an IsoPlate-96 (PerkinElmer). About 350 μg nanoSPA beads for His-tagged proteins (Scintillation Nanotechnologies) equilibrated in 17.5 μl binding buffer were added to reach a final concentration of 5 mg/ml and incubated for 1 h at 4 °C, while gently shaking. [2,8-^3^H]-ATP binding was quantified by a MicroBeta TriLux Wallac 1450 (PerkinElmer) measuring blue light emission of each sample for 2 min (ParaLux mode: high efficiency). Results were background-corrected with samples incubated with 1% (w/v) SDS for 10 min at room temperature. IC_50_ values were determined according to Equation 4, where cpm, T, and U represent bound [2,8-^3^H]-ATP, maximal bound [2,8-^3^H]-ATP in the absence of ATP, and unspecific bound [2,8-^3^H]-ATP, respectively.

(4) $$\text{cpm}=U\;+\;\frac{\left(T-U\right)}{(1+10^{(\left(\log\;{\text{IC}}_{50}-\left[\text{ATP}\right]\times \text{slope}\right)}}$$

### Statistical analysis

Statistical significance analyses were performed using GraphPad Prism 8.0.2. For pairwise comparison statistics, unpaired two-tailed Student's *t* tests with Welch's correction were applied. For multiple comparisons, the parametric Welch's ANOVA test with post hoc Tamhane's T2 test was performed.

## Data availability

All data are accessible from the corresponding author upon reasonable request (R. A.: abele@em.uni-frankfurt.de).

## Supporting information

This article contains supporting information.

## Conflict of interest

The authors declare that they have no conflicts of interest with the contents of this article.
