# Supplementary material for: Interdomain communication in a homodimeric ABC transporter
Source: J Biol Chem. 2024 Jun 5;300(7):107440. doi: 10.1016/j.jbc.2024.107440 (PMC11267003; doi:10.1016/j.jbc.2024.107440)
Supplement: Supporting Information [file mmc1.docx]

Supporting information

**Interdomain communication in a homodimeric ABC transporter**

Katharina-Astrid Lindt^1^, Stefan Frühschulz^1^, Robert Tampé^1^, Rupert Abele^1^*

^1^Institute of Biochemistry, Biocenter, Goethe University Frankfurt, Frankfurt a.M., Germany

*for correspondence: abele@em.uni-frankfurt.de

Running title

Interdomain communication

**Keywords**

ABC transporters; TAPL; interdomain signal transmission; interaction network; allosteric coupling

####
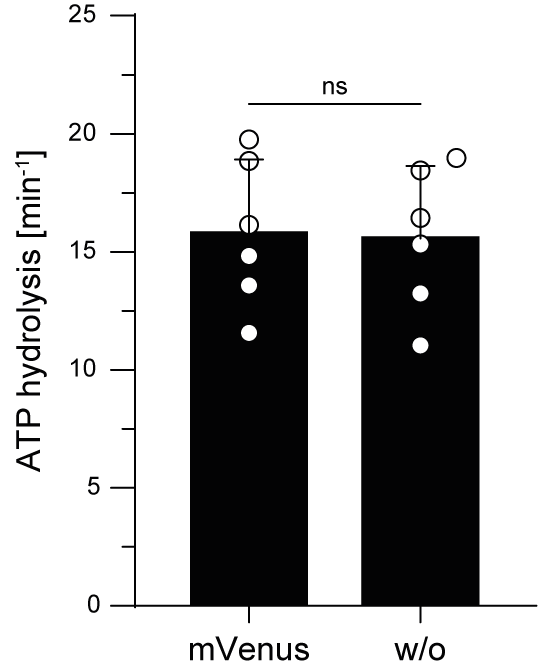


#### Figure S-1 | ATP hydrolysis of coreTAPL is unaffected by the C-terminal mVenus fusion protein

ATP hydrolysis of coreTAPL in the presence and absence of the C-terminal mVenus fusion protein was analyzed with 1 mg/ml proteoliposomes containing TAPL variants (40:1 (w/w) lipid to protein) for 10 min at 37 °C under steady-state conditions (0.3 mM ATP, 3 mM MgCl_2_) (biological replicates, n = 1; technical replicates, N = 3, measured in duplicates). ATP hydrolysis was quantified by autoradiography. Presented data are normalized for equal reconstitution. Data represent the means and standard deviation. Statistical significances were determined using a parametric, unpaired, two‑tailed t‑test with Welch’s correction (ns, non‑significant, P > 0.05; *, P ≤ 0.05; **, P ≤ 0.01; ***, P ≤ 0.001; ****, P ≤ 0.0001).

####
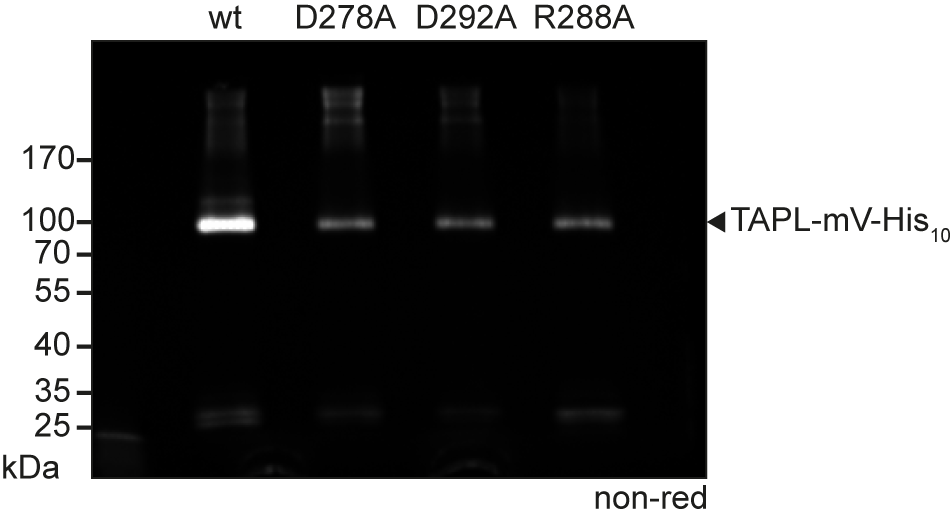


Figure S-2 | Quantification of the mVenus fusion protein of reconstituted TAPL variants in liposomes allows normalization for equal reconstitution

#### TAPL variants were expressed in *Sf21* insect cells, solubilized with DDM and purified by immobilized metal affinity chromatography via a C‑terminal His_10_‑tag. TAPL variants were reconstituted into unilamellar liposomes (40:1 (w/w) lipid to protein) composed of *E. coli* polar lipids and DOPC (7:3 (w/w)). Equal amounts of proteoliposomes were loaded onto a non‑reducing SDS-PAGE, and in‑gel fluorescence of the mVenus fusion protein was quantified at λ_ex/em_ = 480/535 nm.

####
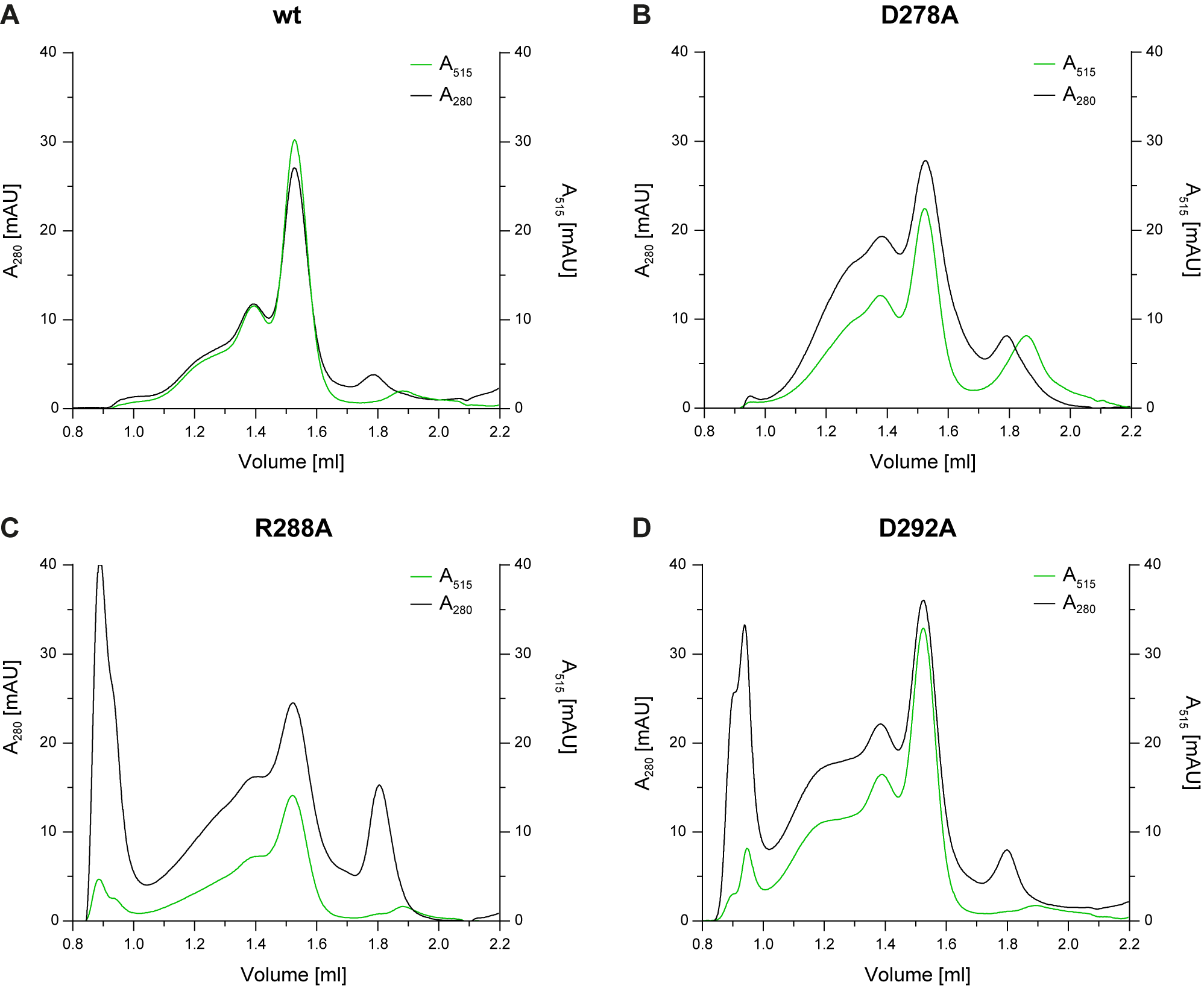


Figure S-3 | Analytical SEC profiles indicate correct folding of all TAPL variants

#### TAPL variants were expressed in *Sf21* insect cells, solubilized with GDN, and purified by immobilized metal affinity chromatography via a C‑terminal His_10_‑tag. 25 µl of GDN‑purified TAPL were injected on a Superose 6 Increase 3.2/300 at 4 °C at a flow rate of 0.04 ml/min. Detectors monitored A_280_ (black line) and A_515_ (green line).

**
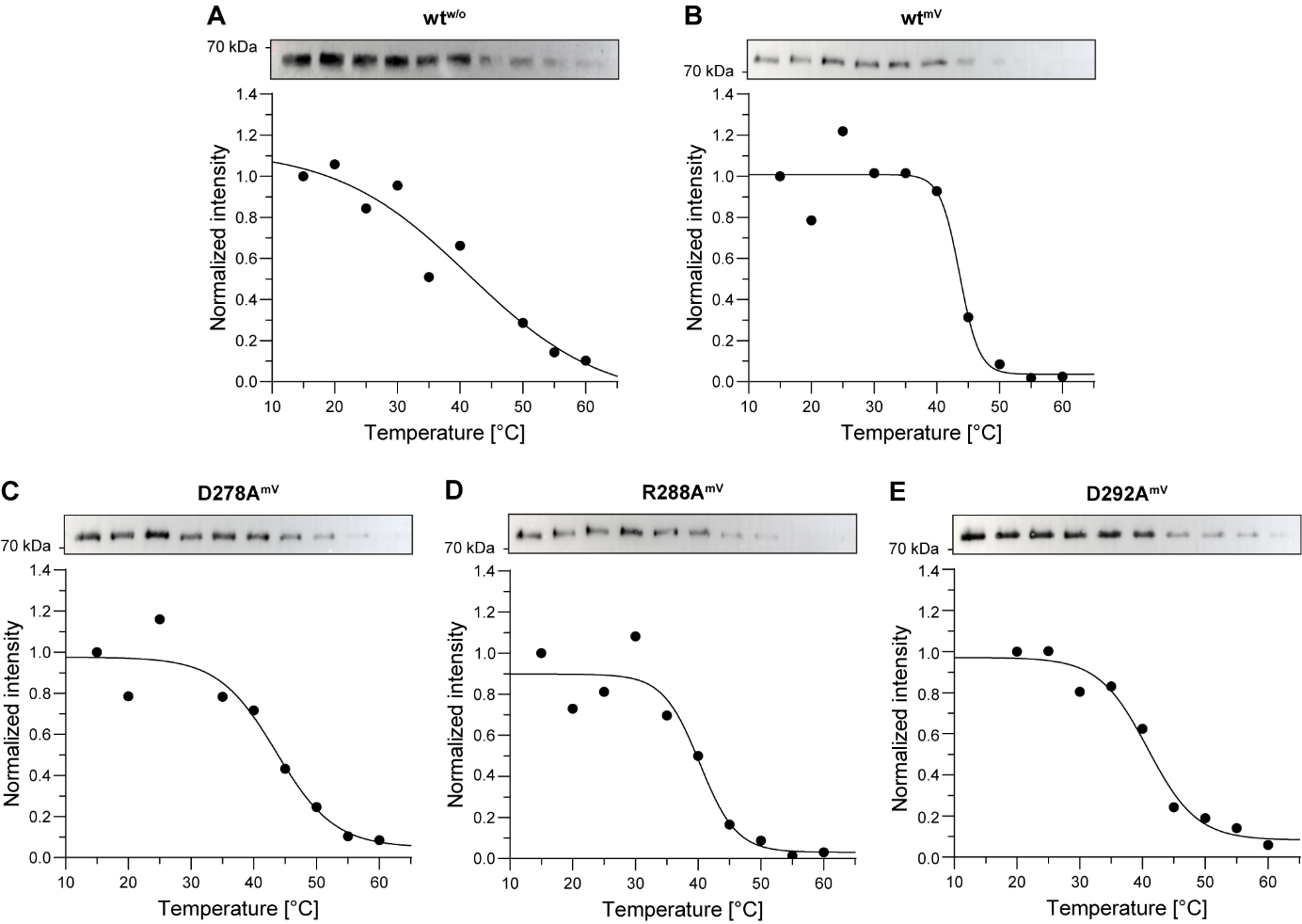
**

#### Figure S‑4 | All TAPL variants show similar thermal stability

#### A‑E GDN-purified TAPL variants were incubated at 15-60 °C for 5 min. After centrifugation, supernatants were loaded onto a non‑reducing SDS-PAGE and immunoblotted using an anti-His-tag antibody. Band intensity of TAPL variants was quantified. Melting temperatures were determined using a sigmoidal dose-response equation with variable Hill slope. Data of two experiments are depicted in Table 1.

####
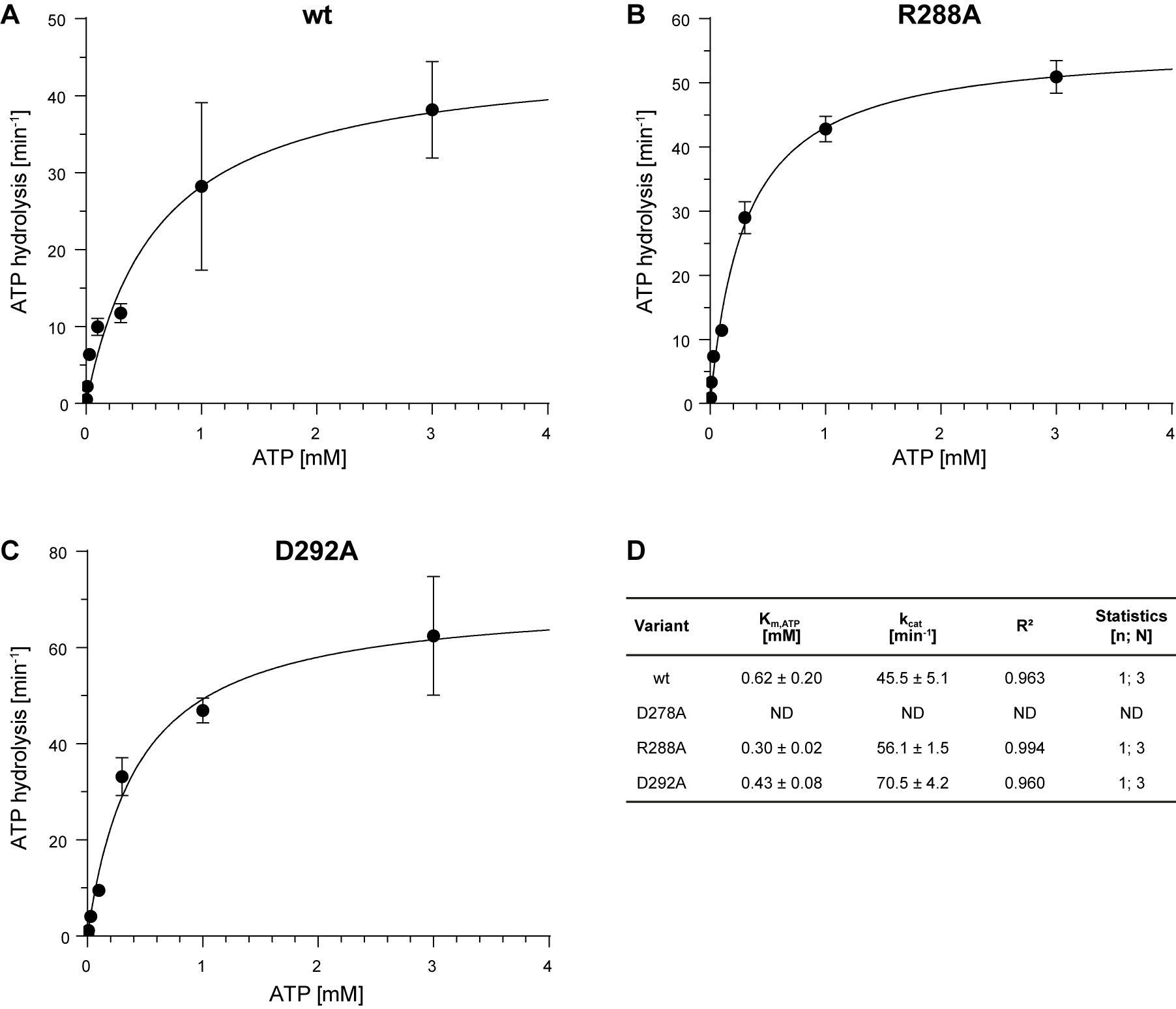


#### Figure S‑5 | ATP-dependent Michaelis-Menten constant K_m,ATP_ of TAPL variants are unaffected by saturating peptide concentrations

#### A-C ATP‑dependent Michaelis‑Menten kinetics of transport‑active TAPL variants in the presence of 150 µM R9L peptide were analyzed with 1 mg/ml proteoliposomes (40:1 (w/w) lipid to protein) and 0.003‑3 mM ATP for 15 min at 37 °C (biological replicates, n = 1; technical replicates, N = 3, measured in duplicates). ATP hydrolysis was quantified by autoradiography. Presented data are normalized for equal reconstitution. Data represent the means and standard deviation. Data were fitted with the Michaelis‑Menten equation. Kinetic parameters and statistics of A-C are listed in D.

Table S-1 | Statistical analysis

#### *ns, non-significant, P > 0.05; *, P ≤ 0.05; **, P ≤ 0.01; ***, P ≤ 0.001; ****, P ≤ 0.0001*

**Figure 2 – Peptide binding**

*Welch’s ANOVA*

| **P value** | **P value**  **summary** |
| --- | --- |
| < 0.0001 | **** |

*Post hoc Tamhane’s T2 multiple comparison test*

| **Comparison** | **Mean 1 ± SD** | **Mean 2 ± SD** | **P value**  **summary** | **P value** |
| --- | --- | --- | --- | --- |
| wt – D278A | 26.42 ± 1.71 | 12.41 ± 2.92 | * | 0.0137 |
| wt – R288A | 26.42 ± 1.71 | 14.94 ± 3.50 | ns | 0.0845 |
| wt – D292A | 26.42 ± 1.71 | 16.15 ± 4.26 | ns | 0.2750 |

**Figure 3 – Peptide transport**

*Welch’s ANOVA*

| **P value** | **P value**  **summary** |
| --- | --- |
| < 0.0001 | **** |

*Post hoc Tamhane’s T2 multiple comparison test*

| **Comparison** | **Mean 1 ± SD** | **Mean 2 ± SD** | **P value**  **summary** | **P value** |
| --- | --- | --- | --- | --- |
| wt – D278A | 26.17 ± 2.06 | 0.11 ± 0.25 | **** | < 0.0001 |
| wt – R288A | 26.17 ± 2.06 | 12.37 ± 1.91 | **** | < 0.0001 |
| wt – D292A | 26.17 ± 2.06 | 10.82 ± 1.12 | **** | < 0.0001 |
| wt – K545A/H699A | 26.17 ± 2.06 | -0.24 ± 0.29 | **** | < 0.0001 |

**Figure 4 – NTP‑dependent peptide transport**

*Welch’s ANOVA*

| **Comparison** | **P value** | **P value**  **summary** |
| --- | --- | --- |
| GTP | < 0.0001 | **** |
| CTP | < 0.0001 | **** |
| UTP | < 0.0001 | **** |
| ITP | < 0.0001 | **** |

*Post hoc Tamhane’s T2 multiple comparison test*

| **Comparison** | **Mean 1 ± SD** | **Mean 2 ± SD** | **P value summary** | **P value** |
| --- | --- | --- | --- | --- |
| **GTP** |  |  |  |  |
| wt – D278A | 100.00 ± 9.06 | -1.26 ± 1.81 | **** | < 0.0001 |
| wt – R288A | 100.00 ± 9.06 | 0.12 ± 0.13 | **** | < 0.0001 |
| wt – D292A | 100.00 ± 9.06 | 0.54 ± 1.03 | **** | < 0.0001 |
| **CTP** |  |  |  |  |
| wt – D278A | 100.00 ± 7.93 | -0.78 ± 1.72 | **** | < 0.0001 |
| wt – R288A | 100.00 ± 7.93 | 0.29 ± 0.46 | **** | < 0.0001 |
| wt – D292A | 100.00 ± 7.93 | 0.18 ± 0.32 | **** | < 0.0001 |
| **UTP** |  |  |  |  |
| wt – D278A | 100.00 ± 15.89 | -1.56 ± 1.71 | *** | 0.0008 |
| wt – R288A | 100.00 ± 15.89 | 0.47 ± 0.55 | *** | 0.0009 |
| wt – D292A | 100.00 ± 15.89 | 0.15 ± 0.17 | *** | 0.0009 |
| **ITP** |  |  |  |  |
| wt – D278A | 100.00 ± 14.30 | -0.45 ± 1.91 | **** | < 0.0001 |
| wt – R288A | 100.00 ± 14.30 | 0.34 ± 0.45 | **** | < 0.0001 |
| wt – D292A | 100.00 ± 14.30 | 0.37 ± 0.53 | **** | < 0.0001 |

**Figure 5 – ATP hydrolysis**

*Welch’s ANOVA*

| **P value** | **P value**  **summary** |
| --- | --- |
| < 0.0001 | **** |

*Post hoc Tamhane’s T2 multiple comparison test*

| **Comparison** | **Mean 1 ± SD** | **Mean 2 ± SD** | **P value**  **summary** | **P value** |
| --- | --- | --- | --- | --- |
| wt – D278A | 14.33 ± 2.69 | -3.67 ± 0.48 | **** | < 0.0001 |
| wt – R288A | 14.33 ± 2.69 | 39.08 ± 6.09 | **** | < 0.0001 |
| wt – D292A | 14.33 ± 2.69 | 12.07 ± 5.21 | ns | 0.7073 |

**Figure 6 – GTP hydrolysis**

*Welch’s ANOVA*

| **P value** | **P value**  **summary** |
| --- | --- |
| < 0.0001 | **** |

*Post hoc Tamhane’s T2 multiple comparison test*

| **Comparison** | **Mean 1 ± SD** | **Mean 2 ± SD** | **P value**  **summary** | **P value** |
| --- | --- | --- | --- | --- |
| wt – D278A | 18.74 ± 3.79 | -5.49 ± 0.11 | **** | < 0.0001 |
| wt – R288A | 18.74 ± 3.79 | 40.72 ± 8.93 | *** | 0.0007 |
| wt – D292A | 18.74 ± 3.79 | 6.77 ± 4.56 | *** | 0.0002 |

**Figure 8 – Substrate-dependent ATP hydrolysis**

*Parametric, unpaired, two‑tailed t‑test with Welch’s correction*

| **Comparison** | **Mean 1 ± SD** | **Mean 2 ± SD** | **P value**  **summary** | **P value** |
| --- | --- | --- | --- | --- |
| wt^w/o^ – wt^R9L^ | 12.14 ± 6.96 | 14.47 ± 2.77 | ns | 0.4713 |
| D278A^w/o^ – D278A^R9L^ | -2.49 ± 1.68 | -3.48 ± 1.51 | ns | 0.3076 |
| R288A^w/o^ – R288A^R9L^ | 12.51 ± 2.87 | 51.02 ± 10.90 | *** | 0.0002 |
| D292A^w/o^ – D292A^R9L^ | 12.22 ± 3.05 | 7.04 ± 5.01 | ns | 0.0610 |

**Figure S1 – ATPase −/+ C-terminal mVenus**

*Parametric, unpaired, two‑tailed t‑test with Welch’s correction*

| **Comparison** | **Mean 1 ± SD** | **Mean 2 ± SD** | **P value**  **summary** | **P value** |
| --- | --- | --- | --- | --- |
| wt^mVenus^ – wt^w/o^ | 15.78 ± 3.13 | 15.57 ± 3.06 | ns | 0.9087 |
